# The design of a user-centred m-health application for caregivers of children undergoing ORL surgery

**DOI:** 10.1093/eurpub/ckac130.043

**Published:** 2022-10-25

**Authors:** R Dobrina, A Cassone, L Brunelli, S Zanchiello, C DeVita, A Starec, L Bicego

**Affiliations:** 1 Healthcare Professions Department, Institute for Maternal and Child Health IRCCS Burlo Garofolo, Trieste, Italy; 2 Department of Medical, Surgical and Health Science, University of Trieste, Trieste, Italy; 3 Innovation and Complex Systems Department, Area Science Park, Trieste, Italy

## Abstract

**Background:**

In the context of common surgical procedures in pediatric otorhinolaryngology (ORL) patients, providing for education to families for hospitalization, surgery, and postoperative home management has been shown to improve peri-operative outcomes. In this regard, the use of Mobile Health Applications (MHA) is increasing. However, for these tools to be needs-appropriate and effective, their development requires a user-centred approach.

**Methods:**

Our study aimed to explore the informational needs and preferences - in terms of features and functionalities - of health care providers and ORL patients’ caregivers (end-users) to inform the development of an MHA supporting ORL peri-operative process effectively. The study was conducted at a 136-bed maternal and child health hospital in Trieste. A user-centred participatory study design was employed, and the methodology steps were informed by the 3 cycles of the Information System Research Framework (Schnall et al., 2016).

**Results:**

The Relevance cycle was performed to better understand the environment as well as end users’ (64 participants) informational needs and desired features for the MHA. Five critical information/education moments of the ORL perioperative period were identified. In the Rigour cycle a literature review was performed to identify further key topics relevant to understanding ORL end-users’ needs and relevant features for the MHA. In the Design cycle the final contents were defined to be displayed on the MHA spread across the 5 identified moments. A randomized controlled trial will then be conducted to evaluate the effectiveness of the MHA compared to standard care.

**Conclusions:**

Triangulation of data sources collected by experts, ORL patients’ caregivers, and healthcare professionals ensured the rigour of the methodology adopted in the study. Moreover, such a MHA user-centred developed MHA favours end-users positive health outcomes and organizational benefits of health services.

**Key messages:**

- Contextualizing, and responding to the health informational needs and intent to use by end users is a critical foundation for the design and adoption of MHA in a maternal and child health hospital.
- To implement MHA quality in the process of children ORL health management and family consumer satisfaction, user-centred design methods are needed and feasible.

